# Editorial: Emerging Fungal Plant Pathogens

**DOI:** 10.3389/fcimb.2021.765549

**Published:** 2021-09-17

**Authors:** Samantha C. Karunarathna, Sajeewa S. N. Maharachchikumbura, Hiran A. Ariyawansa, Belle Damodara Shenoy, Rajesh Jeewon

**Affiliations:** ^1^Center for Mountain Futures, Kunming Institute of Botany, Chinese Academy of Science, Kunming, China; ^2^School of Life Science and Technology, University of Electronic Science and Technology of China, Chengdu, China; ^3^Department of Plant Pathology and Microbiology, College of Bioresources and Agriculture, National Taiwan University, Taipei, Taiwan; ^4^CSIR-National Institute of Oceanography Regional Centre, Visakhapatnam, India; ^5^Department of Health Sciences, Faculty of Medicine and Health Sciences, University of Mauritius, Reduit, Mauritius

**Keywords:** biocontrol agents, climate change, emerging plant diseases, novel fungal pathogens, phylogeny, taxonomy

The occurrence of new and emerging phytopathogenic fungal pathogens is on the rise but has largely been overlooked because of inadequate detection methods (Fisher et al., 2012). Factors associated with such a phenomenon can be attributed to plant pathogens expanding beyond their normal geographic ranges due to globalization and international commerce, adaptive potential, climate and ecological changes as well as modern agricultural practices such as modified land uses and the profuse use of antifungal agents in agricultural practices (El-Sayed and Kamel, 2020). Emerging fungal pathogens are an increasing threat to ecosystems, global health, food security and global economy but remain neglected and understudied despite their potential devastating impact on economically important crops (Fones et al., 2020). These emerging pathogens can act as “true reservoirs” for future disease epidemics, but there are still numerous scientific challenges and research gaps to be resolved as to how these fungal pathogens are transmitted, evolving, adopting novel ecological strategies, switching hosts and causing infections. There is published evidence that common saprophytic fungi belonging to *Cryptococcus*, *Aspergillus* and *Penicillium* species are now emergent as potential plant pathogens. The latter can represent a major threat to staple crops such as rice, wheat, maize and potatoes either during cultural practices or during the post-harvest/storage stages (Alshannaq and Yu, 2017). If these pathogens are not detected and accurately identified in a timely matter and targeted disease-management strategies are not implemented, global food security could potentially be dramatically affected (Fones et al., 2020). To generate and promulgate better scientific insights into this new area of research, we proposed the Research Topic “Emerging Fungal Plant Pathogens”. In this Research Topic, we accepted 10 articles, including 5 reviews and 5 original articles that focus on fungal characterization of emerging plant pathogenic fungi based on polyphasic approaches, their functional roles in diseases, their control methods, taxonomy, phylogeny, and evolution. It is anticipated that this Research Topic will enable plant pathologists to gain better insights into the phytopathogenic lifestyles, identification, phylogeny, host associations and evolution of emerging fungal pathogens. Several authors have contributed papers to this Research Topic and an overview of the scientific content is summarized below.

Gunasinghe et al. presented a comprehensive account of *Neopseudocercosporella capsellae*, the causative agent of white leaf spot of Brassicaceae, as an emerging pathogen. This pathogen was recently reported as causing damage to economically important Brassicaceae crops, including oilseed rape, vegetables, condiment, and forage brassicas. This review discusses the factors affecting the emergence of the disease and efficient disease control measures. Previously, a lack of information on critical aspects of this pathogen’s life cycle limited the development of effective control measures. Gunasinghe et al. confirmed the ability of *N. capsellae* to switch between two morphologies (septate hyphae and single-celled yeast phase) on a range of artificial culture media (*in-vitro*) or *in planta* on the host surface before infection occurs. It was shown that the hyphae-to-yeast transformation occurs through the production of two morphologically distinguishable blastospore (blastoconidia) types (meso-blastospores and micro-blastospores) and arthrospores (arthroconidia).

*Bipolaris sorokiniana* (syn: *Cochliobolus sativus*) is the causal agent of a wide range of cereal diseases. Al-Sadi summarized the latest findings on *B. sorokiniana*, with specific emphasis on genetic, chemical, cultural, and biological control measures. Hazelnut (*Corylus heterophylla*), an important nut crop in China, is reportedly in decline due to the destructive effects of fungal branch canker and dieback. To document the main fungal pathogens from Chinese wild hazelnut, Gao et al. isolated 51 fungi. Three *Cytospora* and two *Diaporthe* species were identified based on morphological observations coupled with multi-locus phylogenetic analyses, and they also provided additional data on three new species, *viz. C. corylina*, *C. curvispora*, and *D. corylicola* while two known species*, C. leucostoma* and *D. eres* are also documented herein.

Thambugala et al. discussed how fungal antagonists play an important role in controlling plant pathogens and diseases. Since modern agricultural practices are shifting towards reducing the usage of chemically synthesized pesticides, various biocontrol methods, strategies and approaches are being used in plant disease management, and fungi are highlighted as one of the successful methods. Powdery mildew caused by *Erysiphe cichoracearum* seriously affects the yield and quality of tobacco leaves, and once it occurs, it often results in substantial economic losses. Jiao et al. investigated the biocontrol efficiency of *Bacillus amyloliquefaciens* YN201732 against *E. cichoracearum*. The results from *in vitro*, spore germination, and greenhouse-pot studies demonstrated that antimicrobial lipopeptides, especially bacillomycin D and fengycin, may contribute to the prevention and control of tobacco powdery mildew. A new fungal species, *Penicillium linzhiense* is isolated and described in Liang et al. with the potential for usage as a biocontrol agent, especially for economically important phytopathogens or emerging pathogens causing diseases on citrus or rice. Recent advancements in the improvement and application of molecular methods for diagnosing widespread and emergent plant pathogenic fungi are discussed in Hariharan and Prasannath. Grasslands are an ecologically and economically important component of the earth’s vegetation, and fungal communities in grasslands play a huge role on the stability of grasslands. Karunarathna et al. addressed the taxonomy, phylogeny and ecology of grassland fungal pathogens and their interactions in grassland ecosystems. This review highlights the importance of understanding the behavior of fungal pathogens in highly diverse grasslands, providing novel insights for controlling diseases in commercial crop fields.

Climate change seriously impacts agricultural practices and food security. It has the potential to negatively affect numerous economically important crops in the future. In addition, perennial crops, such as tea, are particularly vulnerable. Climate change will not only affect crops but also likely affect crop-associated fungal pathogens. Tibpromma et al. predicted how future climatic conditions will impact tea and its associated pathogens.

In summary, this Research Topic with 10 scientific contributions consolidates and expands our knowledge regarding recent advances in plant fungal pathogens and their control measures. To facilitate the effective management of diseases, it is vital to accurately and rapidly detect and identify plant pathogenic fungi, especially ones emerging on economically important crops. In this context, the research articles published herein provide taxonomic information on fungal species supplemented with DNA sequence data to assist future plant pathologists and mycologists better identify similar species.

## Author Contributions

SK, SM and RJ drafted the editorial, and all authors contributed to editorial revision. All authors contributed to the article and approved the submitted version.

## Conflict of Interest

The authors declare that the research was conducted in the absence of any commercial or financial relationships that could be construed as a potential conflict of interest.

## Publisher’s Note

All claims expressed in this article are solely those of the authors and do not necessarily represent those of their affiliated organizations, or those of the publisher, the editors and the reviewers. Any product that may be evaluated in this article, or claim that may be made by its manufacturer, is not guaranteed or endorsed by the publisher.

## References

[B1] Alshannaq A. Yu J. H. (2017). Occurrence, Toxicity, and Analysis of Major Mycotoxins in Food. Int. J. Environ. Res. Public Health 14 (6), 632. doi: 10.3390/ijerph14060632 PMC5486318 28608841

[B2] El-Sayed A. Kamel M. (2020). Climatic Changes and Their Role in Emergence and Re-Emergence of Diseases. Environ. Sci. Pollut. Res. 27 (18), 22336–22352. doi: 10.1007/s11356-020-08896-w PMC7187803 32347486

[B3] Fisher M. C. Henk D. A. Briggs C. J. Brownstein J. S. Madoff L. C. McCraw S. L.. (2012). Emerging Fungal Threats to Animal, Plant and Ecosystem Health. Nature 484 (7393), 186–194. doi: 10.1038/nature10947 22498624 PMC3821985

[B4] Fones H. N. Bebber D. P. Chaloner T. M. Kay W. T. Steinberg G. Gurr S. J. (2020). Threats to Global Food Security From Emerging Fungal and Oomycete Crop Pathogens. Nat. Food 1 (6), 332–342. doi: 10.1038/s43016-020-0075-0 37128085

